# Arthroscopic all-inside repair with suture hook for horizontal tear of the lateral meniscus at the popliteal hiatus region: a preliminary report

**DOI:** 10.1186/s12891-020-3066-2

**Published:** 2020-01-29

**Authors:** Chao-Hua Fang, Hua Liu, Zheng-Lin Di, Jun-Hui Zhang

**Affiliations:** 1 Department of Joint Surgery, the 6th Hospital of Ningbo, No.1059 Zhongshan road, Yinzhou District, Ningbo, 315000 Zhejiang People’s Republic of China; 2 Department of Orthopaedic Surgery, Second Affiliated Hospital, School of Medicine, Zhejiang University, No.88 Jiefang Road, Hangzhou, 310009 People’s Republic of China

**Keywords:** Horizontal tear, Meniscus, Meniscus repair, Popliteal hiatus

## Abstract

**Background:**

Arthroscopic surgery procedures vary depending on the types of meniscus tear, including meniscectomy and meniscus repair. Among the several types of meniscus tear, the horizontal tear of the lateral meniscus at the popliteal hiatus region is a common injury, and its surgical treatment is still inconsistent.

**Methods:**

Between January 2018 and October 2018, 20 patients who underwent all-inside repair with suture hook for the horizontal tear of the lateral meniscus at the popliteal hiatus region were recruited. Any operative complication was recorded, and postoperative MRI scans were conducted at the 6 months. The clinical results were graded based on the scale of the Lysholm knee score preoperatively and at follow-up.

**Results:**

No operative complications were recorded. Postoperative MRIs at the 6 months showed that there was no re-tear for all patients, though signal intensity remained high in T2-weighted MRI in the lateral meniscus for nine cases. The average preoperative Lysholm knee score was 58.6 ± 10.1, which increased significantly to 89.3 ± 7.8 (t = − 11.01, *p* = 0.001) at the last follow-up. Recurrence or aggravation of symptoms was not noted at the final follow-up.

**Conclusion:**

All-inside repair with suture hook may be a good option for the horizontal tear of the lateral meniscus at the popliteal hiatus region which preserves the meniscus; avoids iatrogenic injury on the adjacent popliteal tendon, common peroneal nerve, and inferior lateral geniculate artery.

## Background

Arthroscopic surgery procedures vary depending on the types of meniscus tear, including meniscectomy and meniscus repair. Generally, degenerative changes are reduced at long-term follow-up when normal meniscus tissue is preserved [1]. Among the several types of meniscus tear, horizontal tear is a common injury, and its surgical treatment is still inconsistent. Although the extent of resection is difficult to surgically determine, partial meniscectomy is extensively employed. Historically, some surgeons preferred to remove all tissues to which the tear extends because horizontal tear tends to recur following partial meniscectomy [2]. With this approach, the increased contact area between the femoral condyle and tibial plateau may cause osteoarthritic change and instability over time. Another surgical variant is to resect either the superior or inferior leaf of the horizontal tear [3]. However, with the removal of only one leaf, the remaining leaf is always unstable for the meniscus at the popliteal hiatus region, due to the absence of fixation to the coronary ligament and knee joint capsule [4]. According to a research, regardless of whether inferior leaflet or both leaflet resection was performed for the horizontal medial meniscal tears, femorotibial contact pressure increased and contact area decreased significantly. The peak pressure of the medial compartment in neutral alignment status is 1.95 ± 0.57 Mpa with intact menscus, which can be increased to 2.45 ± 0.87Mpa with single-leaflet resection and 2.54 ± 0.76 Mpa with double-leaflet resection respectively [5]. Meniscus repair is another treatment option and can preserve more meniscus tissue. In order to avoiding suturing the popliteal tendon, the part anterior to hiatus can be repaired by the outside-in technique, and the posterior part undergoes repair of all-inside, outside-in, or inside-out technique. Thus, injury of the popliteal tendon is avoided since the popliteal hiatus is unsutured [6]. Regardless of the suture technique implemented, suturing of the popliteal tendon may cause iatrogenic injury on the adjacent common peroneal nerve and inferior lateral geniculate artery (ILGA) if the nerves have not been exposed and protected [7, 8]. Consequently, surgically repairing a horizontal meniscus tear at the popliteal hiatus region is challenging. Here, we report clinical outcomes after all-inside suture repair with suture hook for the horizontal tear of the lateral meniscus at the popliteal hiatus region. We hypothesized that it can preserve more meniscus tissue and minimize the risk of injury to the adjacent popliteal tendon, common peroneal nerve, and ILGA.

## Methods

**Fig. 1:**
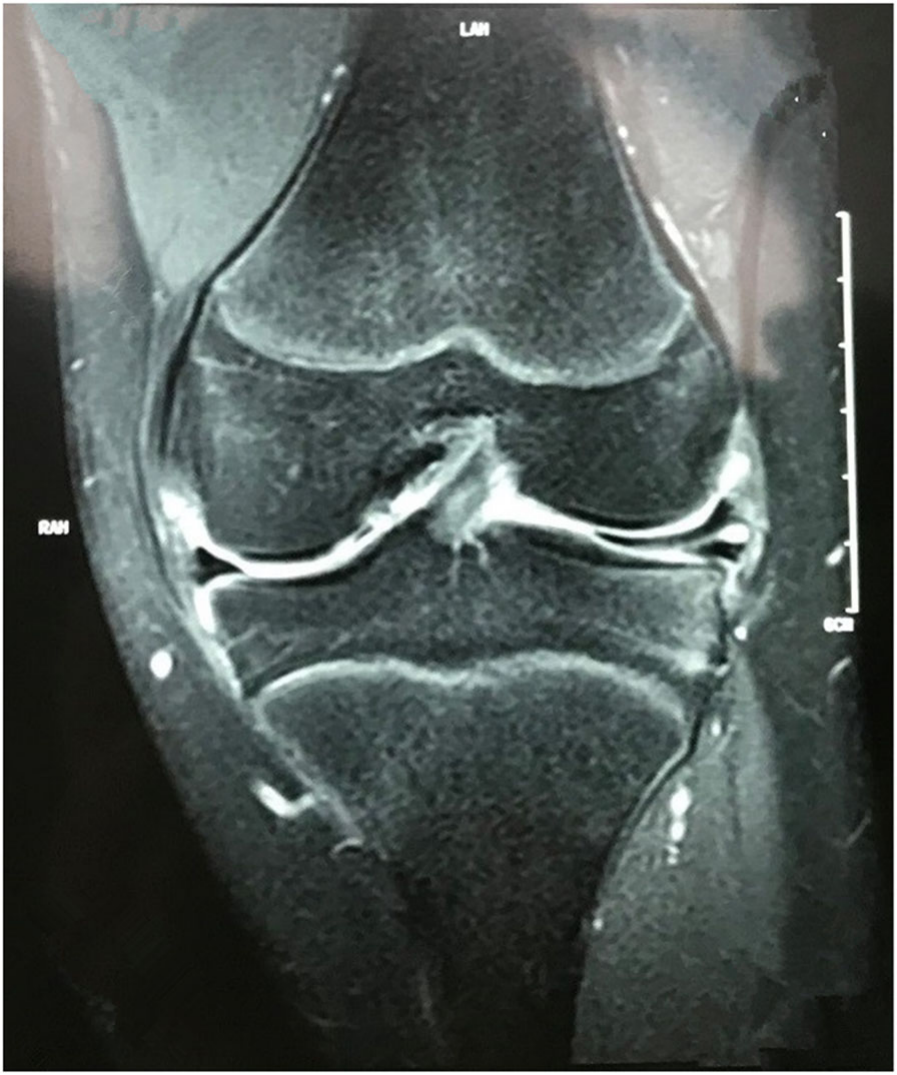
A horizontal tear of the lateral menisci, which is identified with high signal intensity in T2-weighted MRI

3.1 Participants. Between January 2018 and October 2018, a total of 358 patients diagnosed with tear of the lateral meniscus received arthroscopy. The patients who were in accordance with the inclusion criteria were recruited: (1) the physical examination showed positive tenderness at lateral joint gap with the McMurray test; (2) the lateral meniscus tears with or without discoid lateral meniscus were preoperatively found by MRI (Fig. 1). (3) After partial meniscectomy or saucerization was completed, only horizontal tear reach to the popliteal hiatus remained and was required to be treated. The following cases were excluded: concomitant ligament rupture, medial meniscus injury or Outbridge III and IV degree of cartilage injury were found intraoperatively [9]; the cases with the other tears at lateral meniscus that needed to be repaired; and if the laminae of the horizontal tear were too thin or obviously degenerated. Clinical findings and the Lysholm knee score were recorded preoperatively [10].

**Fig. 2:**
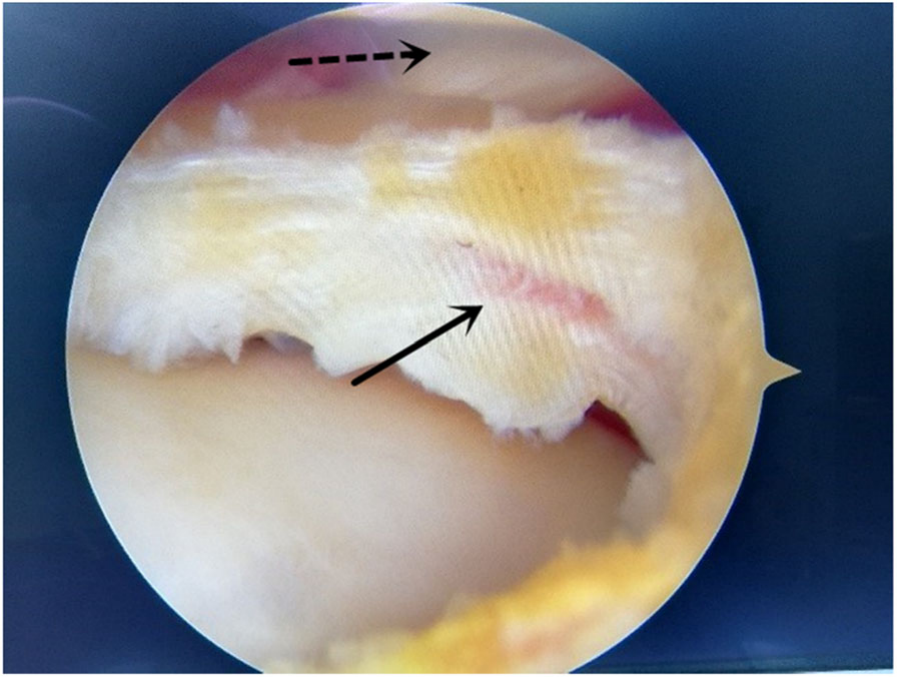
The popliteal tendon (dotted arrow) and a horizontal tear of the lateral meniscus (solid arrow)

**Fig. 3:**
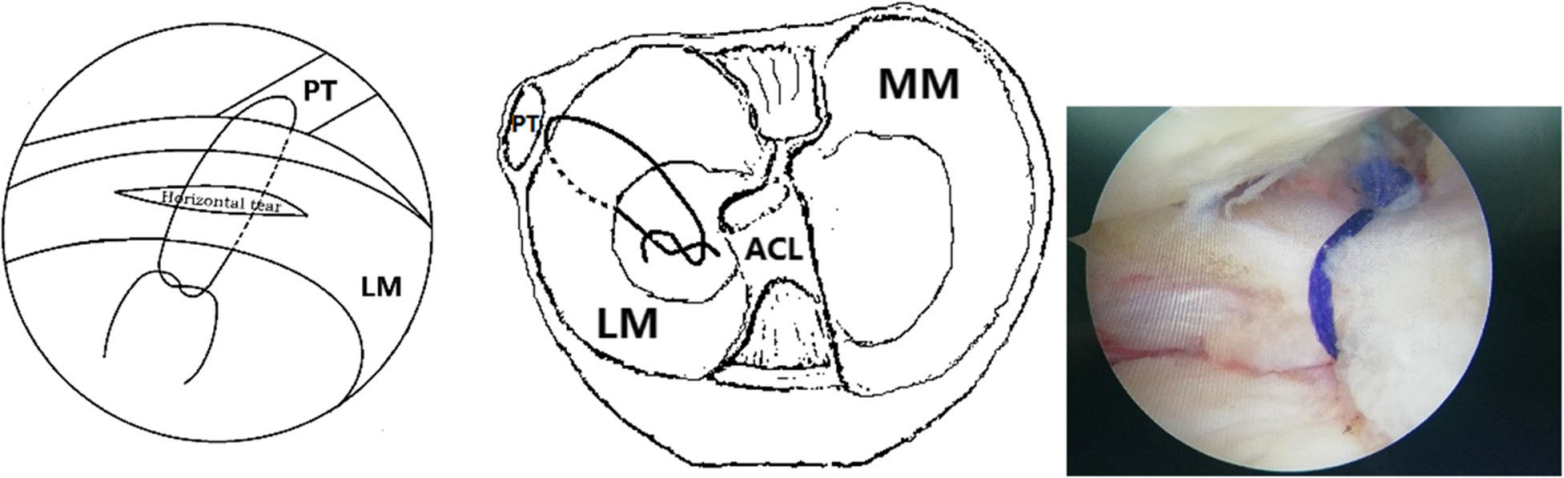
**a** and **b** Procedures of the all-inside suture through the popliteal hiatus from different views. **c** Horizontal tear repaired with a circumferential stitch. PT: popliteal tendon, LM: lateral menisci, MM: medial menisci, ACL: anterior cruciate ligament

3.2 Surgical Technique. A standard supine position was applied for knee arthroscopy. Firstly, standard anteromedial and anterolateral portals were created to inspect the joint. After excluding operative complications at the medial and patellofemoral compartment, the leg was changed to the figure-of-four position. Then debridement, partial meniscectomy, or saucerization was carried out in the lateral meniscus. Usually, the lateral 1/3 width of the meniscus were reserved. The probe was used to examine the horizontal tear at the remnant menisci, which is located at the popliteal hiatus region and extends to the popliteal hiatus in all cases (Fig. 2). Then margins of the meniscus tear were refreshed with a rasp. All these procedures were completed by an arthroscope directly inserted through the anterior lateral portal. The arthroscope inserted through the anterior medial portal was switched and a left curved hook is used for a left knee and vice versa for a right knee. A 25° suture hook (QuickPass Lasso, Low Profile; Arthrex, Naples, FL) loaded with lasso was inserted through the anterior lateral portal, which was used to bypass the popliteal hiatus. The lasso inside the suture hook was pulled out and used as a threader to introduce an absorbable suture (1–0 VICRYL PLUS®, Ethicon, Somerville, NJ). One circumferential stitch with a Tennessee knot and several half stitches were completed to repair the horizontal meniscus tear (Fig. 3). For the tear extend to anterior or posterior positions, Fast-Fix systems® (Smith & Nephew) were used to conduct additional vertical mattress sutures.

3.3 Postoperative Rehabilitation and Clinical Assessment. Postoperatively, the knee was not immobilized with recommended mild motion including strait leg raising, ankle pump and full range of motion of knee in bed. Patients were instructed to avoid squatting, excessive flexion, and sitting with legs crossed for 6 months. Partial weight-bearing was allowed at 3 to 4 weeks, with full weight-bearing permitted at 5 to 6 weeks after surgery. Postoperative clinical results were assessed with the Lysholm knee score 2 weeks and 6 months after surgery. Any operative complications, such as incision or knee joint infection, common peroneal nerve injury, and deep vein thrombosis, were recorded. An MRI was performed again in all the cases to determine the status of recovery at 6 months postoperatively. Strenuous sports activity was allowed once patients have fully recovered.

3.4 Statistical analysis. Statistical analyses were conducted using SPSS 17 software (SPSS Inc., Chicago, IL, USA). Lysholm knee scores were compared using a paired t-test with a *p*-value < 0.05 indicated as statistically significant.

## Results

**Fig. 4:**
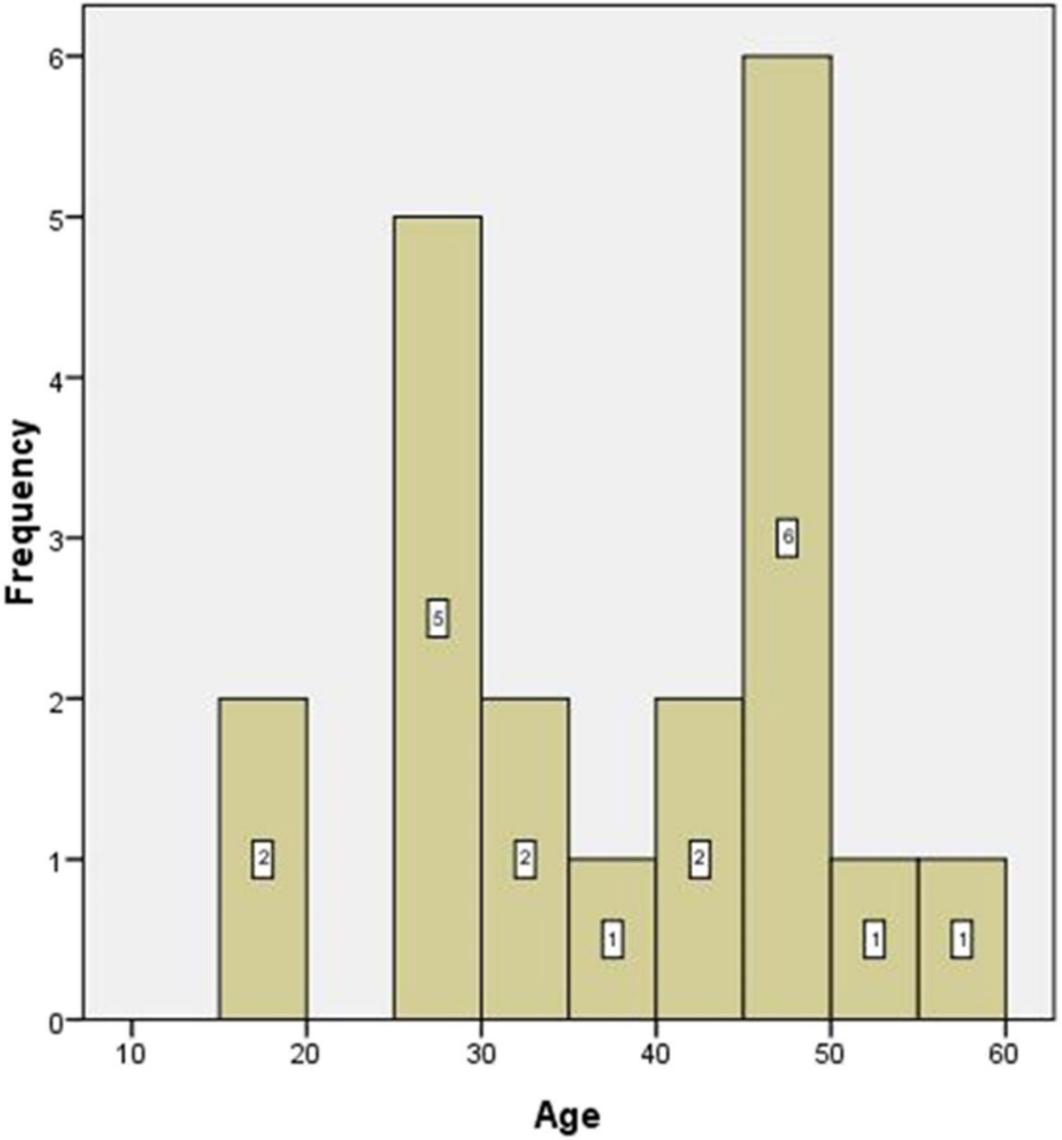
The age distribution of the recruited patients

**Fig. 5:**
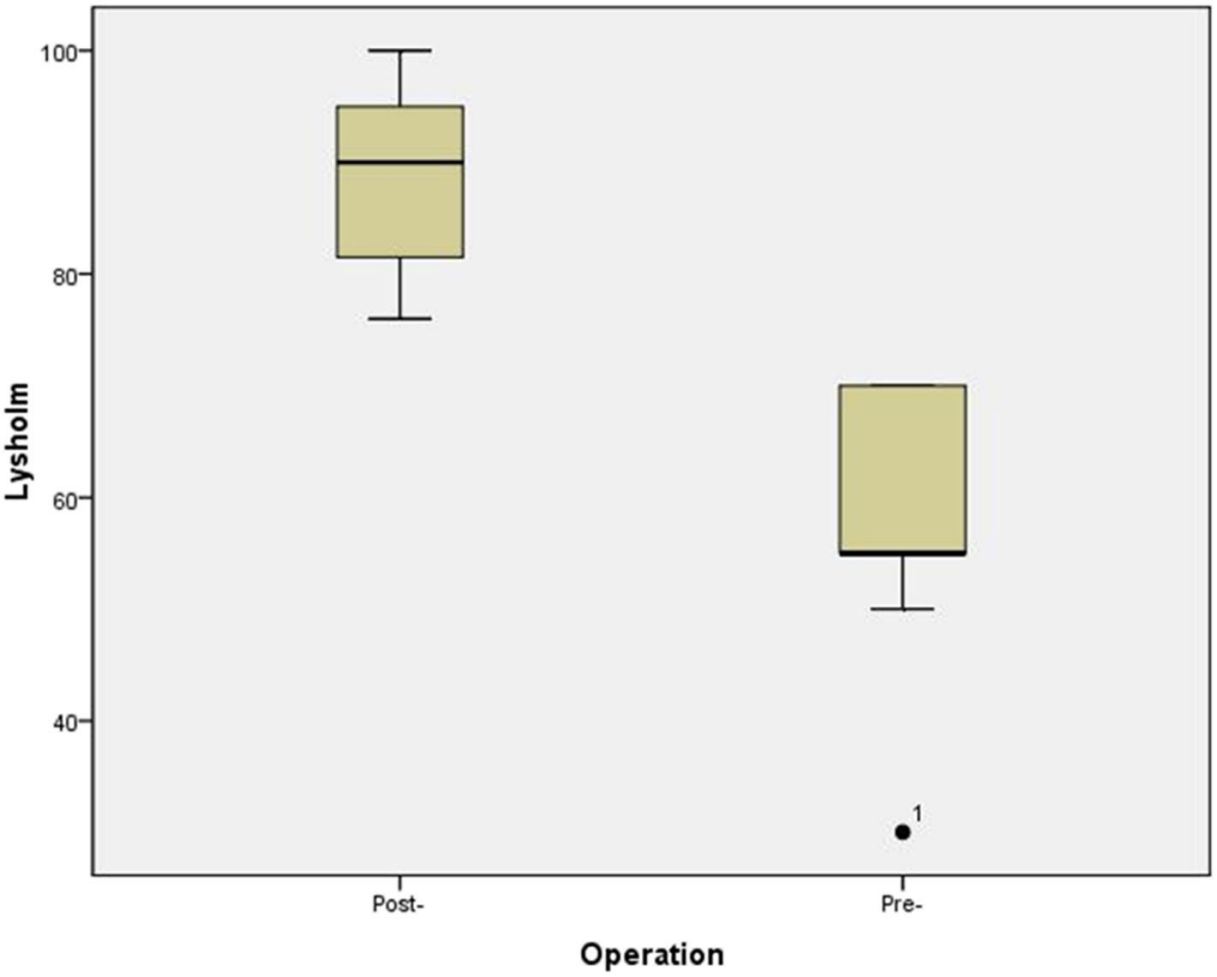
The Lysholm score recorded preoperatively and at follow up

**Fig. 6:**
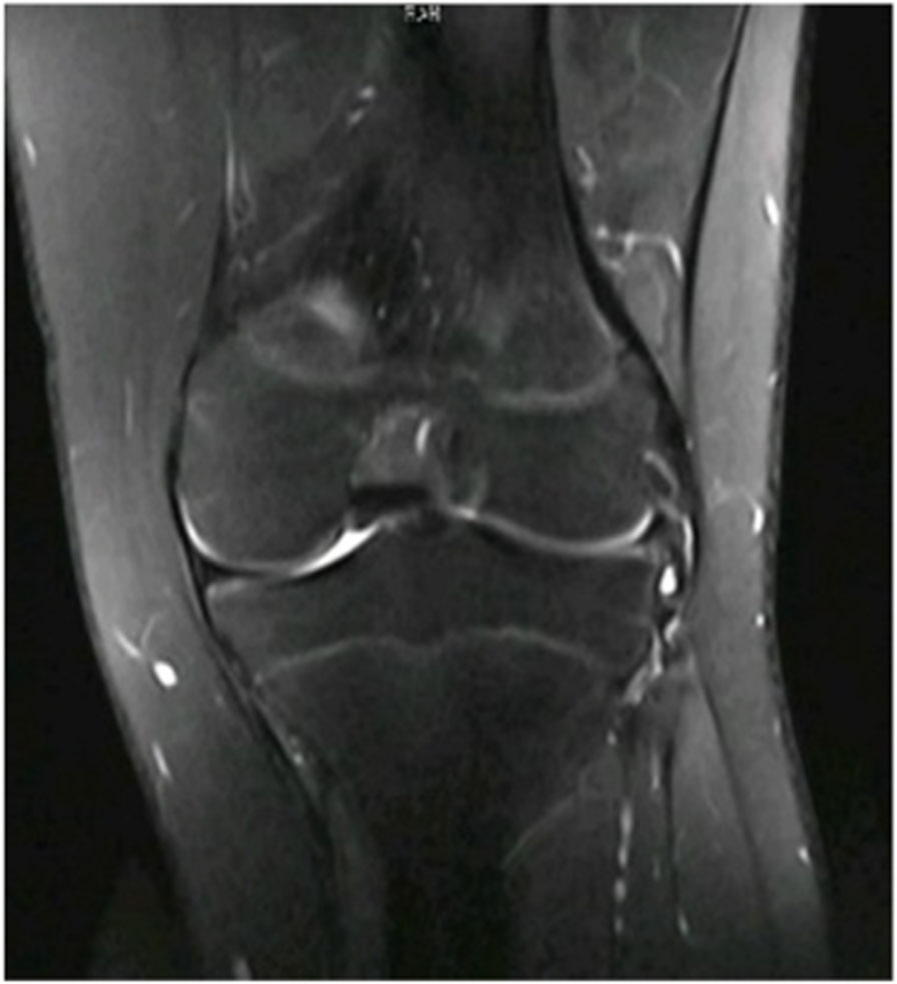
Absence of high signal intensity at the remnant menisci in T2-weighted MRI

In all, 20 cases were finally recruited, including 8 females and 12 males, with mean age 37.2 ± 12.1 years (Fig. 4). In seven cases, lateral discoid menisci were found. Seven cases had a horizontal tear extended to the junction between the body and anterior or posterior horn and Fast-Fix systems® (Smith & Nephew) were used to conduct additional vertical mattress sutures after the circumferential stitches at the popliteal hiatus. This included four cases with one Fast-Fix system® posterior to popliteal tendon, two cases with one Fast-Fix system® anterior to popliteal tendon and one case with one Fast-Fix system® anterior and posterior to popliteal tendon, respectively. The mean follow-up period of all the patients was 11.8 ± 2.1 months (7–15 months), No postoperative complications such as infection or symptomatic venous thromboembolism occurred in any of the patients. The mean preoperative Lysholm knee score was 58.6 ± 10.1, which significantly increased to 89.3 ± 7.8 (t = − 11.01, *p* = 0.001) at the last follow-up (Fig. 5). The postoperative MRI at 6 months showed that there was no re-tear in all cases, though the signal intensity remained high in T2-weighted MRI in the lateral meniscus in nine cases (Fig. 6). Recurrence or aggravation of symptoms was not recorded at the final follow-up.

## Discussion

Although numerous classifications of meniscus tears exist, the one proposed by O’Connor has proved useful. It classified the patterns of meniscus tears into the following categories: (1) longitudinal tears, (2) horizontal tears, (3) oblique tears, (4) radial tears, and (5) variations, which include flap tears, complex tears, and degenerative meniscus tears [11]. Horizontal tears tend to be more common in older patients, with the horizontal cleavage plane occurring from shear, which divides the superior and inferior surfaces of the meniscus. Many flap tears and complex tears begin with a horizontal cleavage component. These are more commonly seen in the posterior half of the medial meniscus or the midsegment of the lateral meniscus.

For the degenerative horizontal tear of the medial meniscus, even no significant difference exist between arthroscopic meniscectomy and non-operative management with strengthening exercises in terms of relief in knee pain, improved knee function, or increased satisfaction in patients after 2 years of follow-up [12]. When considering the risk vs. the benefits of the treatment, conservative therapy is recommended [13]. However, the horizontal tear at the midsegment of the lateral meniscus is most likely located in the popliteal hiatus region. In most instances, either the superior or the inferior leaf is resected to reserve the stable leaf. Based on previous research, the total perimeter of the lateral meniscus was 8.9 ± 0.7 cm, and the length of the hiatus was 1.3 ± 0.1 cm [4]. Without such a distance of the meniscus fixation from the coronary ligament and the knee joint capsule, the remaining superior or inferior leaf is not stable, especially when the tear extends anterior to posterior. On the other hand, a total or partial meniscectomy may result in negative outcomes, including joint alignment, contact pressure, and degenerative changes, which should be avoided [1, 5, 14].

Recently, a case series report recommended a novel arthroscopic all-inside suture technique using the Fast-Fix 360 system (Smith & Nephew, Andover, MA) for repairing horizontal meniscus tears. In this case series, all three patients were athletes and able to resume sport activities a year after surgery. Their postoperative mean Lysholm knee score was 99.7, with absence of pain, complications, and recurrence of meniscus tear. And the MRI signal intensity of all the horizontal tears decreased after surgery, suggesting healing of the repaired tear [15]. A systematic review shows that studies of repaired horizontal cleavage tears show a comparable success rate with repairs of other types of meniscus tears [16]. As a result, repair may be an option, especially for a recent tear and younger patients. When compared with other suturing methods, the all-inside meniscus repair systems have increased in popularity, since they have been shown to be faster and simpler than other methods for meniscus repair, such as meniscus arrows (Bionx Implants, Malvern, PA), Fast-Fix system (Smith & Nephew, Andover, MA), and RAPIDLOC meniscus repair system (Depuy Mitek, Johnson & Johnson, USA) [7]. Usually, sutures are inserted anterior and posterior to the popliteal tendon, with a minimum interval of 1.3 ± 0.1 cm along the length of the hiatus, which may leave the meniscus in the interval unstable. Iatrogenic injury may occur on the adjacent popliteal tendon, common peroneal nerve, and ILGA due to sharp contact of these meniscus repair systems when penetrate them to outside of joint capsule. Use of the outside-in or inside-out technique may injure the common peroneal nerve and ILGA if they have not been protected in advance [7, 8, 17]. The ILGA mainly supplies blood to the lateral meniscus avascular zone adjacent to the popliteus tendon, which is critical for meniscal healing after repair [18]. Insertion of surgical anchors of the meniscus arrows or sutures through the popliteal tendon may lead to iatrogenic injury or irritation to this structure and suture loosening during knee movement [19]. In addition, these repair methods lock the joint capsule with remnant meniscus tissue which was relatively free to the joint capsule, resulting in a reduction of normal movement of the meniscus and the size of popliteal hiatus as well. Both of these outcomes may interrupt the normal biomechanics and kinematics of the lateral knee compartment. However, additional studies are needed to determine and evaluate how the knee joint is affected by these. The current suture method may decrease the risk of complications mentioned above. Firstly, it may preserve more meniscus tissue when compared with a meniscectomy, which benefits the joint alignment and contact pressure. Also, it increases stability of the remnant meniscus which can be achieved if the two leaves heal together, unlike the instability of single laminae alone without fixation from the coronary ligament and knee joint capsule. The most important point is that it is truly all-inside and the intracapsular procedures may avoid injuring the adjacent extracapsular structures [20, 21].

Healing of the reserved meniscus shows a comparable success rate of repair for horizontal cleavage tears; it may be lower at the popliteal hiatus region because of the absence of peripheral vasculature [22]. Postoperative recovery was determined by MRI scan at 6 months because second-look surgery was always rejected in cases without many discomforts. Surgical follow-up was evaluated by the Lysholm knee score. Because the high intensity in MRI may last for a long time, according to Muellner T, the grade III and IV signal alterations can be present on MRI scans in more than 50% of the repaired menisci even after 12 years [23]. Although nine cases still showed a high intensity on T2-weighted MRI in reserved meniscus tissue, all repair cases were considered successful according to the Lysholm knee score. It is suspected that the successful outcomes could be due to the sutures that bind the two separate laminae together and maintain the stability of the lateral menisci. At this time, it is unclear which suture, absorbable or nonabsorbable, is superior. Unfortunately, no nonabsorbable sutures with a similar diameter as the one in Fast-Fix system (Smith & Nephew, Andover, MA) were available at our facility. Another concern is that nonabsorbable sutures remain permanently in the joint cavity. Consequently, an absorbable suture with a suitable diameter (1–0 VICRYL PLUS®, Ethicon, Somerville, NJ) was used in all cases, which is a synthetic, braided suture, made from copolymer (polyglactin-910) of glycolide and lactide, and is absorbed through hydrolysis. Its unique coating and braided feature is easy for smooth passage through tissue, knot tying and knot security. But is need to be worried that its tensile strength of the suture in soft tissue can decrease to 75% after 2 weeks, 50% after 3 weeks and 25% after 4 weeks, and it is completely absorbed within 56–70 days. But it is still unclear about the decline rate of tensile strength in knee joint because of lack of report. Anyway, as indicated in Campbell’s Operative Orthopaedics, the ideal suture material has not been determined, because the human meniscus requires several months to heal completely, the suture selected for meniscal repair should be capable of providing adequate support for this period.Most early reports of meniscal repair advocated the use of an absorbable suture, such as polyglycolic acid (Dexon), polyglactin-910 (Vicryl), or polydioxanone (PDS). As a matter of fact, the mechanical effects of normal joint motion probably cause failure of even nonabsorbable sutures over time [24]. As a result, several movements are banned in 6 months including squatting, excessive flexion, and sitting with legs crossed.

This study still has some limitations. Firstly, we did not evaluate postoperative healing with a second-look surgery which is the current gold standard. MRI follow-up was preferred to avoid complications and pain issues from an additional surgery. Secondly, due to our low subject count and short follow-up period, additional patients need to be recruited with a longer follow-up of up to 2 years, including additional MRI scans for healing assessment. An additional limitation was that this study was uncontrolled. It will be more convincing with comparison groups using an alternative meniscal repair technique or non-surgical management.

## Conclusions

The proposed surgical approach is a good alternative to conduct all-inside technique with suture hook to repair the horizontal tear of the lateral meniscus at the popliteal hiatus region with its advantages of preserving the meniscus; avoiding iatrogenic injury on the adjacent popliteal tendon, common peroneal nerve, and ILGA.

## Data Availability

The datasets used and/or analysed during the current study are available from the corresponding author on reasonable request.

## Abbreviations

ILGA: Inferior lateral geniculate artery
MRI: Magnetic resonance imaging
